# Baseline Characteristics and Results of the Phase 2 COG1201 SHIMMER Study of Zervimesine (CT1812)

**DOI:** 10.1002/alz70859_106890

**Published:** 2025-12-25

**Authors:** James E. Galvin, Mary E. Hamby, Jennifer Iaci, Michael Grundman, Anthony O Caggiano

**Affiliations:** ^1^ University of Miami Miller School of Medicine, Boca Raton, FL USA; ^2^ Cognition Therapeutics, Inc., Pittsburgh, PA USA; ^3^ Cognition Therapeutics, Purchase, NY USA; ^4^ Global R&D Partners, LLC, La Jolla, CA USA; ^5^ Cognition Therapeutics, Inc., Purchase, NY USA

## Abstract

**Background:**

Zervimesine (CT1812) is an experimental oral, small‐molecule drug candidate in development for Alzheimer’s disease and dementia with Lewy bodies (DLB). Zervimesine is designed to protect neurons by preventing the binding of oligomers of pathogenic proteins including β‐amyloid and ɑ‐synuclein.

**Method:**

The COG1201 ‘SHIMMER’ study is the first study to measure tolerability and clinical effects of zervimesine in adults with DLB. The study enrolled 130 individuals with a clinical diagnosis of DLB and MMSE of 18‐27 who were randomized 1:1:1 to receive once‐daily oral doses of zervimesine (100 or 300 mg) or placebo for 26 weeks. Among clinical assessments tools, SHIMMER employed the Neuropsychiatric Inventory (NPI), Alzheimer's Disease Cooperative Study‐Activities of Daily Living (ADCS‐ADL) scale and the Unified Parkinson's Disease Rating Scale (UPDRS) Part III.

**Result:**

Zervimesine‐treated participants experience strong therapeutic responses across behavioral, functional, cognitive, and movement measures in SHIMMER. At the end of the study period, zervimesine‐treated DLB (n=88) patients progressed 86% slower than placebo‐treated patients (n=42) on the NPI, 52% slower on the ADCS‐ADL and 62% slower on the UPDRS Part III. We intend to present for the first time a detailed characterization of the baseline participant characteristics including demographics, baseline scores on outcome measures, concomitant medications, comorbidities and baseline biomarker levels.

**Conclusion:**

These data will allow comparison of the SHIMMER population to prior DLB study populations. SHIMMER results support the potential for zervimesine to slow clinical progression in patients with mild‐to‐moderate DLB. The robust therapeutic response observed across neuropsychiatric, cognitive, motor and functional measures is particularly encouraging. An analysis of participant characteristics will help guide recruitment in future clinical studies of zervimesine and its potential use to treat people with DLB.

Cognition Therapeutics conducted COG1201 with University of Miami Miller School of Medicine and the Lewy Body Dementia Association under a grant from the National Institute of Aging (R01AG071643).

